# A case of musical hallucinations induced by tramadol

**DOI:** 10.1002/npr2.12320

**Published:** 2023-01-19

**Authors:** Tatsu Hase, Norio Yasui‐Furukori, Shigeki Yamaguchi, Kazutaka Shimoda

**Affiliations:** ^1^ Department of Psychiatry Dokkyo Medical University School of Medicine Tochigi Japan; ^2^ Department of Anesthesia and Pain Medicine Dokkyo Medical University School of Medicine Tochigi Japan

**Keywords:** hallucinations, music, opioids, tramadol

## Abstract

**Aim:**

Although tramadol has been suggested to have a higher risk of producing hallucinations than other opioids, reports of musical hallucinations are extremely rare.

**Case Presentation:**

A 72‐year‐old woman came to our department complaining of auditory hallucinations. She had been diagnosed with lumbar spinal canal stenosis associated with herniated and slipped disks. Due to persistent back pain, tramadol was started, and the dose was increased to 300 mg/day. The next day, she began to hear nursery rhymes, songs of the Ministry of Education, and folk songs. The musical auditory hallucinations disappeared with the use of antipsychotics and the discontinuation of tramadol. No relapse of musical auditory hallucinations was observed after the discontinuation of antipsychotics.

**Conclusion:**

Based on the clinical course, we concluded that the auditory hallucinations were musical hallucinations induced by tramadol.

## Abstract 2

Due to persistent back pain, tramadol was started, and the dose was increased to 300 mg/day. The next day, she began to hear nursery rhymes, songs of the Ministry of Education, and folk songs. The musical auditory hallucinations disappeared with the use of antipsychotics and the discontinuation of tramadol.

## Graphical abstract

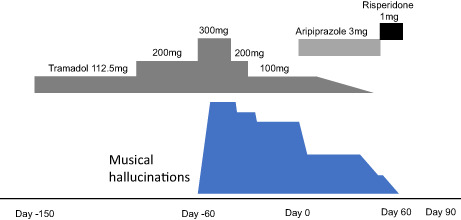

## 1 INTRODUCTION

Musical hallucination is a specific syndrome in which music is heard in the absence of external sound stimulation. Hearing loss, psychiatric disorders (schizophrenia, depression, and obsessive–compulsive disorder), organic brain lesions (brain atrophy, brain tumors, cerebrovascular disease, etc.), epilepsy, and drug addiction have been reported as causal factors for musical hallucinations.^1^, ^2^, ^3^ However, drugs with a high frequency of overall hallucinations, including auditory hallucinations, include Parkinson's disease drugs, benzodiazepines and similar drugs, opioids, anticholinergic drugs such as antidepressants and antipsychotics, and non‐central nervous system‐acting drugs such as ertapenem and voriconazole.^4^ In the present study, we encountered a patient with musical hallucinations caused by oral tramadol. Although tramadol has been suggested to have a higher risk of producing hallucinations than other opioids,^4^ reports of musical hallucinations are extremely rare, and we present this case to demonstrate the need to be aware of the possibility of their occurrence. Written consent to report the case was obtained from the patient.

## 2 CASE PRESENTATION

**FIGURE 1:**
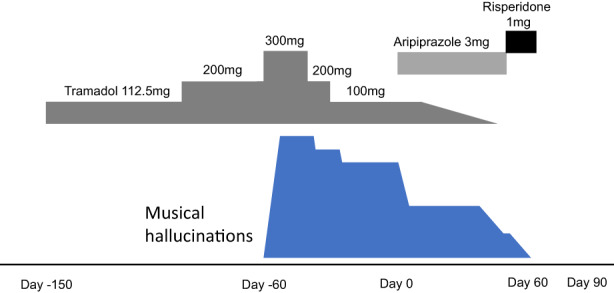
Clinical course of the patient

A 72‐year‐old woman came to our department complaining of auditory hallucinations. Six months earlier, she had developed severe low back pain and numbness in her left lower extremity, which made it difficult for her to walk. She had been diagnosed with lumbar spinal canal stenosis associated with herniated and slipped disks at another hospital. A few days after the onset of her condition, she was started on 75 mg of diclofenac sodium, 112.5 mg of tramadol, and 50 mg of pregabalin. Due to persistent back pain, she was referred to the pain clinic in our hospital 4 months later for block injections. The dose of tramadol was increased to 200 mg, and 1 month later (3 months ago), the dose was increased to 300 mg. The next day (87 days after starting tramadol), she began to hear nursery rhymes, songs of the Ministry of Education, and folk songs, among others, that she had sung in her childhood as part of a chorus. The songs consisted of male and female voices, divided into lower and higher parts, and the same song was repeated approximately 10 times before moving on to another song. Auditory hallucinations appeared mainly during periods of silence, such as before bedtime and while doing housework. Approximately 1 month after the auditory hallucinations began, the patient reported her symptoms to the prescribing physician, and the dose of tramadol was reduced to 200 mg and then to 100 mg 1 week later. After dose reduction, the auditory hallucinations decreased in volume, but the symptoms themselves remained. Magnetic resonance imaging (MRI) of the head and a hearing test were performed to rule out organic lesions, but neither examination showed any abnormalities by a physician General Medicine Department. No ear pain, redness, swelling, ear leaks, plugged ears, tinnitus, dizziness, or cerumen were observed. An electroencephalogram was not performed. There were no signs of psychiatric disease. Mini‐mental examinations showed no cognitive dysfunction with a score of 28. Therefore, the patient was referred to our department and started on aripiprazole 3 mg, which further decreased the volume of auditory hallucinations. Two weeks later, tramadol was tapered off in 25 mg increments and discontinued after 6 days. After discontinuation, the patient's back pain continued without exacerbation with nonsteroidal anti‐inflammatory drugs (NSAIDs) alone. After starting aripiprazole, the patient developed dysrhythmias and a skin rash that were suspected to be drug‐related, so the drug was changed to risperidone 1 mg 42 days later. Ten days later (32 days after the discontinuation of tramadol), auditory hallucinations were almost nonexistent, but dizziness and rhythm disturbances were observed, so risperidone was discontinued 2 weeks after its initiation. After the discontinuation of risperidone, the auditory hallucinations did not recur, and the patient's mental status remained stable. A summary of the clinical course of this case is shown in Figure 1.

## 3 DISCUSSION

In this case, the patient was not suspected of having hearing loss, organic brain lesions, or psychiatric disease. The volume of the musical auditory hallucinations decreased with decreasing drug dose, and musical auditory hallucinations disappeared with the use of antipsychotics and the discontinuation of tramadol (Figure 1). No relapse of musical auditory hallucinations was observed after the discontinuation of antipsychotics, and based on the clinical course, we concluded that the auditory hallucinations were musical hallucinations induced by tramadol. Chronic pain improved with increasing tramadol dose. Furthermore, the musical auditory hallucinations are confined to nursery rhymes, and the auditory hallucinations cause neither pain nor comfort. Therefore, it is unlikely that psychological and social factors are the triggers for these auditory hallucinations.

This is the second case of musical hallucinations induced by tramadol reported in the world. The first case was reported in a 74‐year‐old man with terminal lung cancer who was treated with 200 mg of tramadol.^5^ The musical hallucinations appeared soon after the start of treatment, and the drug was discontinued on the basis of being a suspected side effect of tramadol.^5^ Tramadol discontinuation led to the disappearance of the musical hallucinations 2 days later.^5^ The patient had no organic brain lesions, psychiatric disorders, or history of auditory hallucinations that could have caused the musical hallucinations.^5^ The patient's symptoms resolved immediately after the discontinuation of tramadol, whereas symptom resolution for the patient reported here required a longer period of time and antipsychotic medications.^5^ Being female has been reported as a risk factor for musical hallucinations,^6^ and sex differences may be related to the persistence of symptoms after the discontinuation of tramadol.

A study using a database of all side effects of over‐the‐counter drugs in France reported 4784 adverse drug reactions with tramadol between 1985 and 2013, of which 240 were hallucinations. The odds ratio for a relationship between a drug and the occurrence of hallucinations was high for antiparkinsonian drugs (seven of the top 10 drugs: 17.2–23.1), as well as ertapenem (24.0), scopolamine (17.7), oxybutynin (16.6) and zolpidem (12.9). The odds ratio for tramadol was 6.3, which was second only to that of oxycodone (7.7) among opioids.^4^

Drug‐induced hallucinations may best reflect anticholinergic effects, and it has been suggested that the hallucinations produced by serotonin reuptake inhibitors, in addition to their anticholinergic effects, induce psychiatric symptoms through the serotonin receptor (5‐HT‐3) mediating dopamine release in the ventral striatum.^4^ In addition, the three major opioid receptors, μ, κ, and δ, are all present in the cochlea, inner and outer hair cells, bipolar cells of the spiral ganglion, and interdental cells of the spinal cord, and each has different functions. The functions of these opioid receptors include the regulation of neurotransmitters such as γ‐aminobutyric acid (GABA) and acetylcholine, the decrease in auditory afferent activity through κ receptors, and the increase in auditory afferent activity through μ receptors in the vestibule, suggesting that these opioid receptors play an important role in the adaptive response of the vestibular peripheral organs to metabolic load.^6^ In addition to its opioid action (partial actuation of μ receptors), tramadol has anticholinergic effects through muscarinic receptor antagonism and serotonin reuptake inhibition, which are assumed to induce hallucinations.^4^, ^7^ In addition, the antagonism of GABA receptors and noradrenaline reuptake inhibition of tramadol may also be involved in the appearance of hallucinations.^4^, ^7^ Risk factors for tramadol‐induced hallucinations include advanced age, malignancy, chronic cerebral ischemia or atrophy, psychiatric disorders, and concomitant use of antidepressants and other drugs.^7^ In particular, selective serotonin reuptake inhibitors increase the serotonergic effect of tramadol and inhibit CYP2D6, which is involved in tramadol metabolism.^7^ The response to the onset of hallucinations is to discontinue tramadol or administer an antipsychotic (e.g., haloperidol or risperidone) for a short period of time.^7^

## 4 CONCLUSION

While tramadol has been reported to have a higher risk of producing hallucinations than other opioids, reports of musical hallucinations such as those, in this case, are very rare. Although infrequent, tramadol should be suspected as the causative agent when musical hallucinations occur, and appropriate action should be taken.

## AUTHOR CONTRIBUTIONS

TH, NFY, SY, and KS were involved in the clinical investigations. TH and NYF wrote the manuscript. TH, NYF, and KS were involved in the literature review and revisions. All authors read and approved the final manuscript.

## CONFLICT OF INTEREST

Kazutaka Shimoda has received research support from Novartis Pharma K.K., Dainippon Sumitomo Pharma Co., Astellas Pharma Inc., Meiji Seika Pharma Co., Ltd., Eisai Co., Ltd., Pfizer Inc., Otsuka Pharmaceutical Co., Ltd., Daiichi Sankyo Co., and Takeda Pharmaceutical Co., Ltd., and honoraria from Eisai Co., Ltd., Mitsubishi Tanabe Pharma Corporation, Takeda Pharmaceutical Co., Ltd., Meiji Seika Pharma Co., Ltd., Janssen Pharmaceutical K.K., Shionogi & Co., Ltd., Dainippon Sumitomo Pharma Co., Daiichi Sankyo Co., and Pfizer Inc. The funders did not have any role in data collection or in the study design, analysis, decision to publish, or preparation of the manuscript. The remaining authors declare that they have no competing interests to report.

## ETHICS STATEMENT

Approval of the Research Protocol by an Institutional Reviewer Board: The ethics committee of the School of Medicine at Dokkyo Medical University determined that there was no need to review this case.

Informed Consent: Written informed consent was obtained from the parent for the publication of this case report.

Registry and the Registration No. of the Study/Trial: Not available.

Animal Studies: Not available.

## Data Availability

Data sharing is not applicable to this article as details of the case cannot be disclosed in accordance with the Personal Information Protection Law.
